# NEGATIVE SOCIAL SUPPORTS AND DEPRESSIVE SYMPTOMS AMONG OLDER ADULTS: A CROSS-LAGGED ANALYSIS

**DOI:** 10.1093/geroni/igz038.594

**Published:** 2019-11-08

**Authors:** Nicholas Cone, Gina Lee, Peter Martin

**Affiliations:** Iowa State University, Ames, Iowa, United States

## Abstract

The purpose of the study was to examine how negative social supports and depressive symptoms affect older adults over time. A subsample of participants (N = 3,084) from the Health and Retirement Study was used in this study. Summary scores for each negative social supports (spouse, children, family members, and friends) and the Center for Epidemiologic Studies Depression Scale (CES-D) were used to conduct two cross-lagged regression analyses for each negative social support type from waves 2010 and 2014. Covariate variables for this study included gender, years of education, self-report of health, and age. Results were computed for two age groups (i.e., 65 to 79, and 80+). Results from both age groups indicated high stability for negative social supports and depressive symptoms from waves 1 to 2. The younger age group showed no significant cross-lag or interaction effects when stabilities were included or excluded in the analyses. However, in the older group, wave 2 negative child and family member social support was predicted by wave 1 depression scores. Moreover, the older age group showed significant interaction effects of age by CESD scores on negative child and family member social supports. In conclusion, initial depressive symptoms predict higher negative social supports in children and family members at a second time point in the older age group. Future research could examine whether depressive symptoms continue to predict negative social supports in new waves. In addition, other factors, such as loneliness, or anxiety, may provide further understanding into older adults’ negative social supports.

